# Use of a Pedicled Vascularized Ulnar Nerve as a Long Graft for Complete Brachial Plexus Palsy in Adults to Restore Elbow Flexion: Should this Practice Continue?

**DOI:** 10.1055/a-2694-8871

**Published:** 2025-09-22

**Authors:** Camille Echalier, Jean Noël Goubier

**Affiliations:** 1 Nerve and Brachial Plexus Surgery Institute, Paris, France; 2 Clinique Bizet, Paris, France; 3 Clinique Nollet Paris, Paris, France; 4 Institut de la Main, Clinique Bizet, Paris, France; 5 SOS Main la Francilienne, Hôpital Privé Paul D'Égine, Champigny sur Marne, France

**Keywords:** brachial plexus, nerve graft, vascularized ulnar nerve, allograft

## Abstract

Restoring elbow flexion is a priority in adults with complete brachial plexus palsy. If the nerve root is not avulsed, a graft can be placed between the existing root and the musculocutaneous nerve. The aim of this study was to evaluate the outcomes of using vascularized ulnar nerve grafts in this context. Our case series consisted of 17 male and 3 female patients (mean age of 31 years) presenting complete brachial plexus palsy after a motorcycle accident. A graft at the C5 or C6 root on the musculocutaneous nerve was done in all patients using a pedicled vascularized ulnar nerve to restore elbow flexion at a mean of 5 months after the accident. At a mean follow-up of more than 3 years, elbow flexion was graded as M4 in six patients and between M0 and M2 in the other 14 patients on the Medical Research Council scale. None of the patients had M3 strength. While the results of long grafts using a vascularized ulnar nerve are disappointing in this case series, they are consistent with previous publications. Encouraging results have only been reported with short grafts (<10 cm), which can rarely be used with supraclavicular lesions. For this reason, we currently prefer using a sural nerve graft or nerve transfer, when possible, to restore elbow flexion in adult patients with brachial plexus injuries.

## Introduction

Restoring elbow flexion is a priority in adults with complete brachial plexus palsy. If the upper nerve roots are not avulsed, a graft can be used to restore flexion. 1 Currently, the preferred technique uses the sural nerve. 2 This long graft, however, does not have a feeder artery to ensure its survival with a high risk of central necrosis. 3 For this reason, vascularized nerve grafts have been introduced in 1976, by Taylor and Ham. They were the first to describe using vascularized grafts to improve the outcomes of long nerve grafts, aiming to reduce the likelihood of necrosis of the interposed nerve graft. 1 The aim of this study was to evaluate the outcomes of using pedicled vascularized ulnar nerve grafts to restore active elbow flexion in adults with complete brachial plexus palsy.

## Materials and Methods

This retrospective case series consisted of 20 men who had complete brachial plexus palsy secondary to a motorcycle accident. The mean patient age was 31 years (15–60). The patients were operated a mean of 5 months (2–7) after the accident. All patients underwent a magnetic resonance imaging beforehand to confirm the upper cervical nerve roots were not avulsed.

With the patient under general anesthesia, the cervical area was explored to confirm the C5 or C6 nerve root was available for grafting. When a root was available, a vascularized ulnar nerve harvested from the ipsilateral arm was used a graft on the motor portion of the musculocutaneous nerve. The vascularized graft was 19 cm long, on average (14–20).

**Fig. 1:**
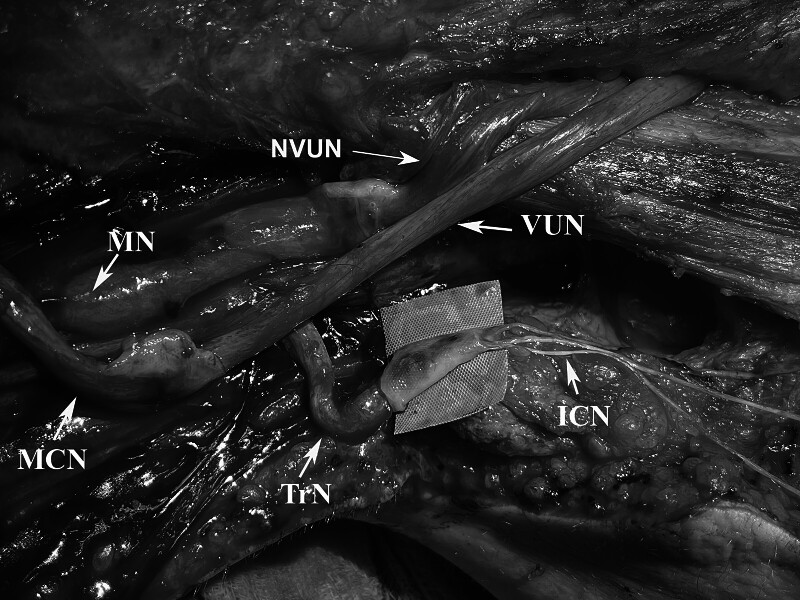
Intraoperative photos (right upper limb). —of distal suturing of a vascularized ulnar nerve graft (UNG) on the musculocutaneous nerve (MC) to restore elbow flexion. —of the transfer of 3 intercostal nerves (ICN) on the long head of triceps nerve (LHTN) to restore elbow extension. —of a nonvascularized ulnar nerve graft remnant (NVUN) being transferred on the median nerve (MN) to restore finger flexion.

In two patients, since another root was available for grafting, a nonvascularized graft (the remaining ulnar nerve) was added between C6 and the median nerve trunk ( Fig. 1 ).

**Fig. 2:**
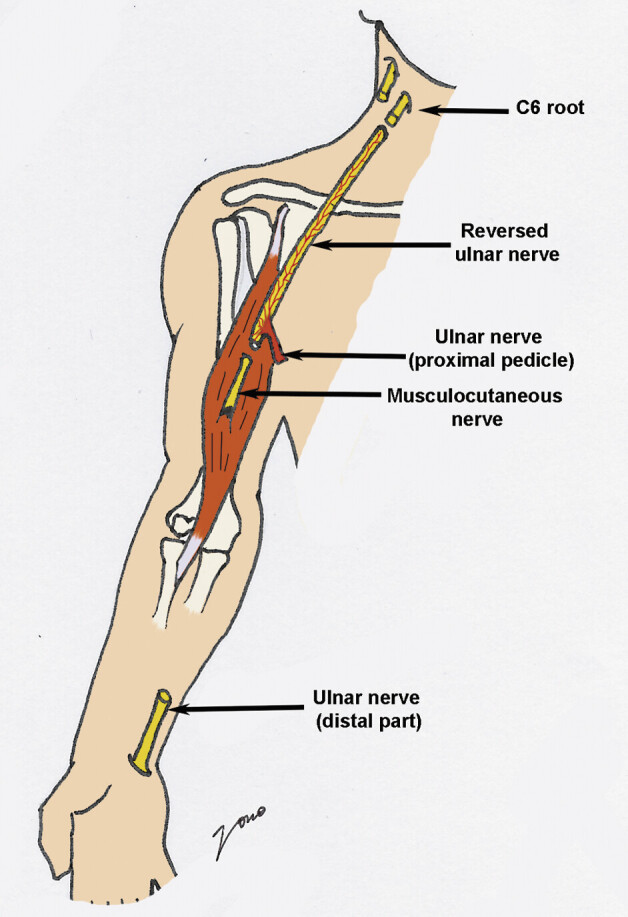
Schema of the vascularized ulnar nerve graft technique. The ulnar nerve is severed at the wrist and then turned around its pedicle, the distal part becomes proximal and is sutured to the root, and the proximal part becomes distal and is sutured to the musculocutaneous nerve.

The vascularized graft was turned around its vascular pedicle and slipped into a subcutaneous tunnel until the cervical region, where it is sutured to the target nerve root ( Fig. 2 ). Using a microscope, epiperineural suturing was done and supplemented by fibrin glue. The patient's arm was immobilized for 6 weeks; rehabilitation was initiated to mobilize the joints and reduce the risk of stiffness.

## Results

**Fig. 3:**
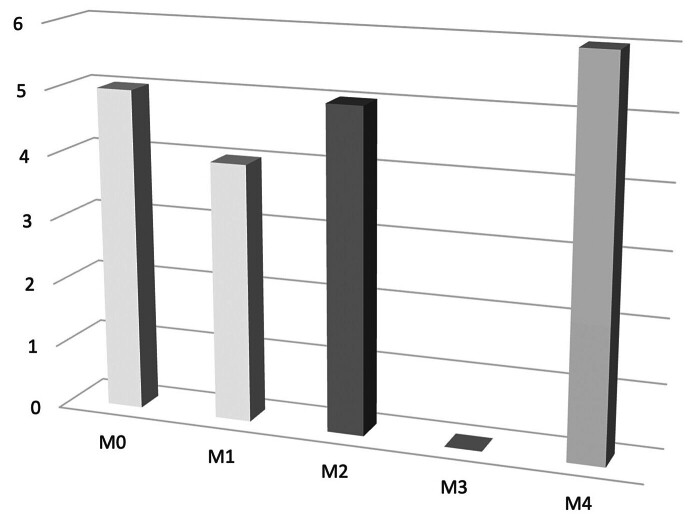
Graph of motor recovery after ulnar vascularized graft for elbow flexion (according the classification of British Medical Council: M0: No muscle activation; M1: Trace muscle activation, such as a twitch, without achieving full range of motion; M2: Muscle activation with gravity eliminated, achieving full range of motion; M3: Muscle activation against gravity, full range of motion; M4: Muscle activation against some resistance, full range of motion; M5: Muscle activation against examiner's full resistance, full range of motion.

The mean follow-up was 38 months (16–120). Six patients recovered M4 elbow flexion strength on the Medical Research Council scale ( Fig. 3 ). Five patients had M2 strength, 4 had M1, and 5 had M0. None of the patients had M3 strength. On average, recovery of elbow strength took 17 months (10–22). None of the patients had persistent pain due to pseudo-Tinel sign sensations during percussion of the cervical area ( Table 1 ).

**Table 1:** Patient data summary

	Age (y)	Sex	Time before surgery (mo)	Ruptured roots	Avulsed roots	Grafted root to MCN nerve	Vascularized ulnar nerve length (cm)	Elbow flexion (BMC scale)	Other procedures
Patient 1	27	M	4	C5C6	C7T1	C5	20	M4	SAN to SSN/ICN to LHTN
Patient 2	29	M	2	C5T1		C5	20	M4	Shoulder fusion/ICN to LHTN/ FL Transfer
Patient 3	16	M	4	C5C6	C7T1	C5	20	M4	SAN to SSN/ICN to LHTN
Patient 4	30	M	4	C5C6	C7T1	C6	19	M4	SAN to SSN/ICN to LHTN
Patient 5	23	M	7	C5C6	C7T1	C5	14	M2	SAN to SSN/ICN to LHTN
Patient 6	35	M	6	C5T1		C5	16	M2	C6 median (NVUN) (shoulder fusion refused)
Patient 7	54	F	2	C5C6	C7T1	C6	20	M1	SAN to SSN/ICN to LHTN
Patient 8	17	M	6	C5C6	C7T1	C5	19	M2	C6 median (SG)/SSN grafting/ICN to LHTN
Patient 9	38	M	2	C5C6	C7T1	C5	20	M0	Trapezius transfer/ICN to LHTN
Patient 10	60	M	6	C5	C6T1	C5	17	M0	SAN to SSN
Patient 11	15	M	5	C5C6C7	C8T1	C6	20	M4	C7 median/shoulder fusion/ICN to LHTN
Patient 12	35	M	3	C5C6	C7T1	C5	19	M1	SAN to SSN/ICN to LHTN
Patient 13	18	F	3	C5C6	C7T1	C5	20	M2	C6 median (SG)/shoulder fusion
Patient 14	35	M	5	C5C6C7	C8T1	C5	19	M4	C6 median (SG)/shoulder fusion
Patient 15	32	F	5	C5C6C7	C8T1	C5	20	M1	C6 median (SG)/shoulder fusion refused
Patient 16	27	M	6	C5T1		C5	19	M0	C6 median (SG)/shoulder fusion
Patient 17	18	M	6	C5C6C7	C8T1	C5	18	M0	C6 median (NVUN)/SSN recovery/ICN to LHTN
Patient 18	31	M	5	C5T1		C5	20	M1	Shoulder fusion
Patient 19	19	M	5	C5T1		C5	19	M2	Shoulder fusion
Patient 20	27	M	6	C5C6	C7T1	C5	20	M0	SSN to SAN

Abbreviations: F, female; FL, fascia lata; ICN intercostal nerve; LHTN, long head of the triceps nerve; M, male; MCN, musculocutaneous nerve; NVUN, nonvascularized ulnar nerve; SAN, spinal accessory nerve; SG, sural graft; SSN, suprascapular nerve.

## Discussion

The results in terms of muscle strength are poor in our series, with only 30% of M4 results. These results are comparable with those reported in the literature. As a matter of fact, in a case series of eight patients with complete brachial plexus palsy, Bertelli et al. did a C5 root graft onto the musculocutaneous nerve using a vascularized ulnar graft. At more than 2 years' follow-up, six patients had achieved M2 strength only in their biceps. 4 The results reported by Birch et al. 5 appear better, likely because their grafts were shorter (14 cm) than ours since they were sutured onto secondary trunks (given that patients had a retroclavicular injury), which led to faster muscle reinnervation and therefore, better motor recovery. After placing a vascularized ulnar nerve graft between the C5 root and the median nerve, Chang et al. reported that 75% of their 25 patients had M3 finger flexion strength, 6 which is a poor functional result.

It is difficult to explain why the results with this technique are inadequate. The vascularization provided by the accompanying artery may not be sufficient to vascularize the entire nerve after its distal end is cut. Thrombosis after pedicle or artery manipulation is another potential explanation, as is spontaneous thrombosis of the extended artery after its distal end is cut. Questions about the suturing's effectiveness are natural when nerve surgery fails. However, the diameter of the ulnar nerve corresponds exactly to that of the torn nerve roots, making the graft and root anatomically compatible. Also, microsurgical suturing is done in a comfortable and reproducible anatomical environment. Lastly, the repairs are sufficiently permeable, since 13 of our patients had achieved M1 to M4 strength and nerve regrowth was occurring based on the Tinel sign, although it did not reach the motor end-plates for sufficient recovery to M3.

Our conclusion that the nerve root was graftable from a macro- and microscopic perspective can be brought into question and may be another reason for failure. Here again, several studies that did not confirm the nerve root was available for grafting based on trimmed nerve specimens examined by histopathology still reported satisfactory results with shorter grafts. Lastly, none of the well-known patient-specific factors that limit nerve regrowth, such as smoking were present in our case series. 7

The lack of a control group with conventional sural grafts is a limitation of our study. However, there are many published studies describing acceptable, but sometimes inconsistent results with this type of graft with no more than 55% of M3 to M4 active elbow flexion, 8 even though the use of a sural nerve remains the most commonly used technique for brachial plexus reconstruction in most international teams. 2

The 3-year follow-up period appears to be sufficient. In fact, motor recovery can take up to 2 years after this type of surgery. 5 The time elapsed between the accident, and the surgery impacts recovery. All the patients in our case series were operated within 5 months on average, a time frame in which the prognosis is best. 9 Chronic denervation of the ulnar nerve due to an initially complex brachial plexus injury can also explain the lower effectiveness of the denervated graft, 10 we believe this is the primary reason for these results.

Excessive regrowth related to graft vascularization and the magnitude of intraneural connections in the ulnar nerve may explain the worse results for this type of graft, as shown by Bertelli et al. 11 While the radial or sural nerve has been used as vascularized grafts they are currently not used for brachial plexus injuries. 12 13 Using a nonvascularized allograft has not yet been evaluated in adult brachial plexus injuries, although some authors have published encouraging results for the repair of injuries to the large peripheral nerve trunks. 14

## Conclusion

While the principle is attractive from an intellectual point of view, the outcomes of using long vascularized ulnar nerve grafts do not yield consistently satisfactory results when used to restore elbow flexion in adults with complete brachial plexus palsy. We now use these graft only when a nerve transfer cannot be done, or the sural nerve cannot be used as a free graft. The use of a vascularized sural nerve for long grafts in the management of long grafts in brachial plexus palsy could be a suitable solution involving the use of a healthy nerve with sufficient vascularization to avoid graft necrosis. A clinical study should be proposed to confirm this hypothesis.
